# Using interviewer random effects to remove selection bias from HIV prevalence estimates

**DOI:** 10.1186/1471-2288-15-8

**Published:** 2015-02-05

**Authors:** Mark E McGovern, Till Bärnighausen, Joshua A Salomon, David Canning

**Affiliations:** Harvard Center for Population and Development Studies, 9 Bow Street, Cambridge, MA 02138 USA; Department of Global Health and Population, Harvard T.H. Chan School of Public Health, Boston, MA 02115 USA; Wellcome Trust Africa Centre for Health and Population Studies, University of KwaZulu-Natal, Mtubatuba, South Africa

**Keywords:** Selection Bias, HIV Prevalence, Missing Data, Heckman Selection Models, Random Effects Estimation

## Abstract

**Background:**

Selection bias in HIV prevalence estimates occurs if non-participation in testing is correlated with HIV status. Longitudinal data suggests that individuals who know or suspect they are HIV positive are less likely to participate in testing in HIV surveys, in which case methods to correct for missing data which are based on imputation and observed characteristics will produce biased results.

**Methods:**

The identity of the HIV survey interviewer is typically associated with HIV testing participation, but is unlikely to be correlated with HIV status. Interviewer identity can thus be used as a selection variable allowing estimation of Heckman-type selection models. These models produce asymptotically unbiased HIV prevalence estimates, even when non-participation is correlated with unobserved characteristics, such as knowledge of HIV status. We introduce a new random effects method to these selection models which overcomes non-convergence caused by collinearity, small sample bias, and incorrect inference in existing approaches. Our method is easy to implement in standard statistical software, and allows the construction of bootstrapped standard errors which adjust for the fact that the relationship between testing and HIV status is uncertain and needs to be estimated.

**Results:**

Using nationally representative data from the Demographic and Health Surveys, we illustrate our approach with new point estimates and confidence intervals (CI) for HIV prevalence among men in Ghana (2003) and Zambia (2007). In Ghana, we find little evidence of selection bias as our selection model gives an HIV prevalence estimate of 1.4% (95% CI 1.2% – 1.6%), compared to 1.6% among those with a valid HIV test. In Zambia, our selection model gives an HIV prevalence estimate of 16.3% (95% CI 11.0% - 18.4%), compared to 12.1% among those with a valid HIV test. Therefore, those who decline to test in Zambia are found to be more likely to be HIV positive.

**Conclusions:**

Our approach corrects for selection bias in HIV prevalence estimates, is possible to implement even when HIV prevalence or non-participation is very high or very low, and provides a practical solution to account for both sampling and parameter uncertainty in the estimation of confidence intervals. The wide confidence intervals estimated in an example with high HIV prevalence indicate that it is difficult to correct statistically for the bias that may occur when a large proportion of people refuse to test.

**Electronic supplementary material:**

The online version of this article (doi:10.1186/1471-2288-15-8) contains supplementary material, which is available to authorized users.

## Background

Estimates of HIV prevalence from serologic testing in nationally representative household surveys have been considered the “gold standard” in developing countries [1]. However, HIV testing participation rates in these surveys may be low. For instance, in the Demographic and Health Surveys (DHS), participation in HIV testing ranges from 97% for women in Rwanda in 2005, to 63% for men in Malawi in 2004 and in Zimbabwe in 2005 [2]. In the DHS, respondents are generally asked if they will consent to a blood test after completing a verbal interview [3].^a^ Although non-participation can result from either not being contacted for interview, or refusing consent to test for HIV after the interview, the latter reason for non-participation is typically much more common [4]. Therefore, in this paper we focus on correcting for missing data arising from refusal to test. If HIV prevalence is different among individuals who test and those who do not test, ignoring missing HIV data may lead to biased estimates of population prevalence. Typical approaches to impute the HIV status of respondents who did not test assume that non-participation is missing at random (MAR), or missing at random conditional on observed covariates. In past studies, this approach has generated estimates of population prevalence that are very similar to estimates based only on the subset of respondents who participated in testing [4, 5]. However, the assumption that there are no unobserved variables that are correlated with both HIV status and consent to test may be unrealistic. Longitudinal data show that a person’s belief about his or her HIV status may be related to actual status, and may influence the likelihood of consenting to an HIV test [6–9]. If this is the case, the missing data can be described as “non-ignorable”, and there will be selection bias in conventional population prevalence estimates based on an incorrect assumption of data missing at random [10]. An additional problem with imputation based approaches is that they are likely to result in confidence intervals which are too narrow because they ignore the uncertainty surrounding the unknown relationship between testing and HIV status, which needs to be estimated. The implicit assumption in these imputation approaches that this correlation is zero with certainty is likely to be violated in many cases.

Several methods have been proposed to account for the selection problem when missing data are non-ignorable, including the use of longitudinal data [7, 8, 11] and alternative testing procedures [12]; however these approaches are not applicable to existing cross-sectional datasets, such as the DHS. Therefore, an attractive alternative to imputation and other similar methods (for example, inverse-probability weighting), is to use a model that explicitly accounts for the selection process, such as that proposed by Heckman [13]. This approach allows us to obtain asymptotically unbiased and consistent estimates of HIV prevalence, even in the presence of unobserved variables which influence non-participation, such as belief about HIV status [14]. By accounting for both non-participation and HIV status equations explicitly in the model (individuals first chose whether to participate, and it is only conditional on this choice that we observe their HIV status), the key benefit of this method is that we do not require the assumption of data missing at random. In the 2007 Zambia DHS survey, estimated HIV prevalence among men who refused consent to test was found to be 53% using the sample selection model, but only 12% in an imputation model [15]. These results seem plausible when viewed in terms of the longitudinal evidence which indicates that those who refuse to test are substantially more likely to be HIV positive [6–9]. The same method has been used to produce selection-corrected HIV prevalence estimates for other African countries, as well as at Health and Demographic Surveillance Sites [2, 16–18]. These findings provide evidence that selection on unobserved characteristics may be an important source of bias in some existing estimates.

However, there are a number of drawbacks associated with the standard implementation of Heckman-type selection models in the current literature. Because of the way the selection and HIV status equations are typically specified, it is often not possible to use this approach because the models can fail to converge due to collinearity problems. Additionally, the small sample properties of the standard models can be poor. Finally, the standard errors which are typically reported in applications of Heckman-type selection models are too narrow because they do not account for parameter uncertainty in the model estimation. The goal of this paper is to introduce a new method which corrects for each of these limitations. Our method is based on an interviewer random effects model, which can be easily implemented in all surveys, even in the problematic cases where HIV prevalence or selection is either very low (<10%) or very high (>90%). In what follows, we explain the drawbacks associated with the standard Heckman-type model, and outline how our new method corrects for these issues. Finally, we illustrate our approach using household survey data from Zambia and Ghana.

## Methods

### Existing Heckman-type selection models for estimating HIV prevalence

Following the framework adopted by Bärnighausen and colleagues, we model consent to test for HIV for person *i* with interviewer *j* as the observed outcome arising from a latent variable that can be interpreted as the propensity to consent to testing [15]:
1

where *s*_*ij*_ is a dummy indicator variable for agreeing to test, *x*_*ij*_ are observed characteristics, *z*_*j*_ are interviewer effects, *u*_*ij*_ is random error, and  is an unobserved latent variable. The equation for the HIV status *h*_*ij*_ of individual *i* with interviewer *j* is:
2

where  is again a latent variable,^b^ and *ϵ*_*ij*_ is an error term. The measured outcomes, consent to test, *s*_*ij*_, and HIV status, *h*_*ij*_, are individual level variables. The main independent variables for both equations, *x*_*ij*_, include predictors at the individual and household level. These socioeconomic, geographic, and demographic characteristics are derived from the DHS survey data. In order to provide asymptotically unbiased estimates, Heckman-type selection models additionally require a selection variable which predicts consent to test, but not HIV status [19]. In this case, we use interviewer identity, *z*_*j*_, as the selection variable, as it is plausibly unrelated to HIV status. Interviewer allocation is most likely a function of survey design, and therefore should not be related to respondents’ characteristics, such as HIV status. Therefore, we include interviewer identity as an additional predictor of consenting to test for HIV. In DHS surveys with nested HIV surveys, once a person has been contacted, the interviewer provides her with information about HIV testing and offers her an HIV test during an informed consent process. The identity of the interviewer who sought consent from that participant is recorded in the dataset as an anonymized code, one for each respondent.^c^

Previous papers have also used the identity of interviewers as a selection variable that is correlated with consent to test but is assumed to be unrelated to HIV status [13, 16, 17]. For those who are absent and not contacted for interview, day of first contact with the household is an alternative selection variable when survey design allows for follow-up attempts to contact missing participants [13].^d^ Better interviewers may have a personality type (for example the ability to show empathy for the interviewee), or relevant experience, which increases their participation rates. This assumption is testable, and interviewer identity is highly correlated with consent in our data. Interviewers are not randomly assigned to participants in DHS surveys, but are assigned to specific regions and tend to be matched to respondents by sex and language. However, sex and language (as well as a rich set of other participant characteristics) are recorded in the DHS datasets, and once they have been controlled for, the interviewer assignment is likely random and uncorrelated with participants’ HIV status. Assuming the selection variable is valid (interviewer identity predicts consent to test but not HIV status), Heckman-type selection models will provide asymptotically unbiased estimates, even when the data are not missing at random [14]. Simulation studies have confirmed that this method provides reasonable estimates of HIV prevalence in large samples under the assumption that, conditional on observed participant characteristics, interviewer identity is uncorrelated with HIV status [20].

In the standard approach, which we refer to as a fixed effects model, and which has previously been used to adjust for missing data in some HIV surveys [15], the selection variable (interviewer identity) takes the form of a series of binary indicator variables in the selection equation, which take the value 1 if the individual was interviewed by that interviewer, and 0 otherwise. For interviewers *j* = 1 … *J*, there will therefore be *J* - 1 indicator dummy variables *z*_1_ … *z*_*J* - 1_, with the *J*th being the omitted category. This Heckman selection approach is an individual level model because the selection and HIV status equations are measured at the individual level (a binary indicator for whether the respondent consented to test, and a binary indicator for whether that respondent was HIV positive among those who consented to test). However, the interviewer indicator variables (fixed effects) take the same value (1 or 0) for all respondents who were interviewed by the same interviewer. In this sense, the model is hierarchical: respondents are nested within interviewers.

As the dependent variables are binary, we adopt a bivariate probit Heckman-type selection model, which models both consent to test and HIV status equations simultaneously in a maximum likelihood framework [21, 22]. The error terms in these equations (*u*_*ij*_, *ϵ*_*ij*_) are assumed to be drawn from a bivariate normal distribution, each with mean zero, variance 1, and correlation parameter *ρ* = *corr* (*u*_*ij*_, *ϵ*_*ij*_). This approach of modelling consent and HIV status jointly allows us to adjust for dependence between selection into testing and HIV status [13].

The key parameter in the model is *ρ*. If *ρ* = 0, there is no correlation between testing and HIV status once observed variables have been controlled for, and imputation of the HIV status of those who do not test based on their observed characteristics is possible [4, 5]. If *ρ* ≠ 0 however, conditional on observed characteristics testing is correlated with HIV status, and the probability of being HIV positive among those who refuse to test will be different from the prevalence rate among people with similar observable characteristics who do test. The key issue in the model is therefore to find robust estimates of *ρ*. Regarding inference, the DHS surveys are generally carried out within fixed strata representing urban and rural areas of each region. Within each stratum, a cluster of households is randomly selected from within a set of possible primary sampling units (PSU), usually defined by a preceding census [23]. All of our models use robust standard errors which adjust for this complex sample design by clustering at the PSU level. We also weight our estimates of HIV prevalence to match the national population using the household level weights provided by the DHS.

### Limitations of existing approaches

There are three main limitations to the use of Heckman-type selection models which include fixed effects for each interviewer as the selection variables which predict consent to test but not HIV status. First, these fixed effects are often not identified for interviewers who conducted few interviews, or who have only successes or failures in consent.^e^ Fixed effects may also not be identified for interviewers who only interview people of a particular language in a particular region, as this can result in their fixed effects (*z*_*j*_) being collinear with these interviewee characteristics (*x*_*ij*_). These problematic interviewer fixed effects are typically pooled to a common value by creating a new category for these interviewers, which also includes all interviewers who complete less than 50 interviews. The assumption of a common value for the interviewer effect on consent in these cases is difficult to justify. In addition, even when interviewer fixed effects are formally identified, this identification may be weak and lead to lack of convergence in model estimates.

Second, while maximum likelihood estimators are asymptotically unbiased and consistent, their small sample properties can be poor [24, 25]. In a simulation study, the finite sample bias for the maximum likelihood estimate in a related recursive bivariate probit model was particularly large when the true probability of the outcome was near the boundary of zero or one [26]. This bias occurs because the maximum likelihood estimator selects the most likely parameter value (the mode of the likelihood function), and not the expected value of the parameter. When the likelihood function is uni-modal and symmetrical, the maximum likelihood estimate is usually unbiased, at least asymptotically. This case will arise, for example, when estimating regression parameters in the linear model with Gaussian errors where maximum likelihood estimates are identical to ordinary least squares estimates. However, when the parameter space is bounded, and the true parameter is near the boundary (in our case, *ρ* = ±1), the likelihood function is usually highly skewed and its mode and mean can be very different. Rates of non-convergence in bivariate probit models can be high when this occurs [27], and the maximum likelihood estimate can be highly implausible in these cases (for example, predicting that everyone who declines to test is HIV positive or HIV negative).^f^ This problem is clearly illustrated in our analysis of the Ghanaian data where HIV prevalence is low.

Finally, the analytic standard errors for the HIV prevalence estimates which are typically reported in applications of Heckman-type selection models are too narrow because they do not account for regression parameter uncertainty in constructing the joint model of selection and HIV status. Bootstrap standard errors have been shown to provide reliable inference in the context of bivariate probit models, particularly when the number of observations is low, and either the probability of the outcome occurring or the probability of selection is high or low [26]. Unfortunately, the bootstrap procedure is difficult to implement with the fixed effects approach as the number of interviewees per interviewer will vary with each bootstrap sample, and different sets of interviewer fixed effects become unidentified in each iteration. Alternative parametric simulation approaches require strong assumptions on the correlation between unobserved error terms [2]. In addition, when the maximum likelihood estimate of ρ is at the boundary of the parameter space, the assumptions required for asymptotic normality of the maximum likelihood estimator are no longer met, and inference is non-standard. In particular, the usual properties of the bootstrap approach fail [28, 29].

### New interviewer random effects methodology

We account for these three issues by proposing a random effects approach with a bias correction for cases which are difficult to estimate due to low or high rates of non-participation or HIV prevalence. Instead of introducing a fixed effect for each interviewer into the selection equation, we assume that interviewer effectiveness is a random effect. Where data have a hierarchical structure, researchers often face the choice between a model based on random effects and a model based on fixed effects. Our approach for estimating HIV prevalence can be viewed in terms of this framework. The fixed effects approach requires us to estimate a separate parameter for each interviewer (a series of binary indicator variables), some of which may not be identified. While the random effects approach has the usual cost of requiring the assumption that the interviewer effects are drawn from some distribution (typically a normal distribution), this model has the advantage that fewer parameters are required, and it is therefore more parsimonious (and potentially more efficient as a result). The random effects approach also generally requires the assumption that the random effects are uncorrelated with the individual level covariates, but this assumption can be relaxed by including the average characteristics of each interviewer’s interviewees in the model [30, 31].

The random effects approach allows us to estimate interviewer effects for all interviewers, even those which are not identified in the fixed effects method. Using a regression model, we can predict the interviewer’s position in the distribution of success in eliciting consent to an HIV test, and include this information in the selection equation as a single continuous variable. There are two approaches to estimating these random effects. The first is to use a hierarchical probit for consent with interviewers at the group level. The random effects can then be estimated directly from this model. The alternative is to calculate the random effects as the average of the error terms for each interviewer in a probit model for consent. We have found almost identical results from both methods, however we prefer the latter approach for practical reasons because it is much less time consuming.

Our procedure is therefore as follows. We first estimate the interviewer random effects from the selection equation only (stage one), then include these constructed parameters as our exclusion restriction in a Heckman-type model (stage two). We also include the average characteristics of an interviewer’s interviewees in the selection equation to capture potential differences in interviewee attributes across interviewers [30, 31]. Because our random effects are a single continuous variable, we can estimate the interviewer parameters using this method in cases in which it is difficult to identify and estimate fixed effects. As a consequence, we avoid arbitrary pooling of interviewer effects and allow straightforward bootstrapping to obtain corrected confidence intervals for our HIV prevalence estimates.

We have tested whether including the mean characteristics of the interviewer’s interviewees are required in the model to account for potential correlation between interviewers and the average characteristics of their interviewees. We regress whether a respondent consented to test for HIV on the control variables used in the model (*x*_*ij*_), the interviewer fixed effects (*z*_*j*_, binary indicator variables for whether the respondent was interviewed by that interviewer), and the average values of the control variables for that interviewer (). We then test whether the coefficients on  are jointly equal to 0. For both Zambia and Ghana, our tests reject this null hypothesis, indicating that the  are necessary to avoid bias in the estimation [32]. Therefore, we include them in the model for Zambia and Ghana. However, there may be other contexts where the  are not necessary, and excluding them could be more efficient. We therefore we suggest a two-stage process: first, testing the relevance of the mean interviewee characteristics for consent; second, including them in the selection model along with the interviewer random effect, but only if they are in fact relevant for consent. If not, the interviewer random effects could be used as the selection variable without this correction.

Our cluster bootstrap procedure is performed by resampling at the level of the primary sampling unit (PSU), reflecting the two stage sampling design adopted by the DHS [23]. As respondents are nested within PSU clusters, it is important to account for this dependence when estimating variance parameters [33]. Therefore, with each bootstrap replication we re-calculate the interviewer random effects. However, there are alternative approaches to bootstrapping in hierarchical models [34], and, as we describe above, it is also possible to conceptualize respondents as being nested within interviewers in our model. Therefore, we have also estimated cluster bootstrap models where we resample at the interviewer level. We find very similar confidence intervals in this analysis.

**Figure 1:**
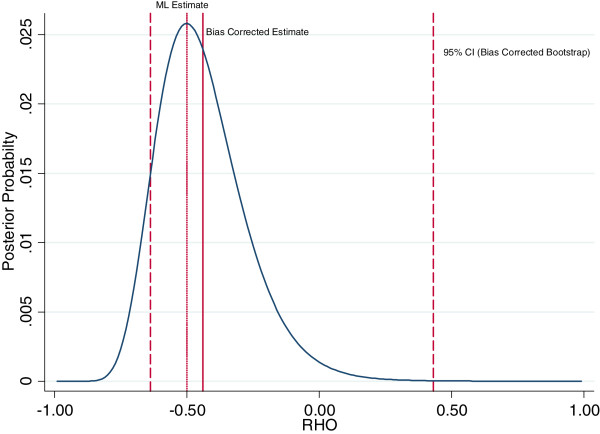
**Posterior probability distribution for the correlation between HIV Testing and HIV Status in Zambia 2007 (Men).** Graph shows the posterior probability distribution for the correlation between testing and HIV status *ρ* = *corr*(*u*_*ij*_, *ϵ*_*ij*_), calculated using a selection model with a flat prior probability distribution over the interval [-1,1], and interviewer random effects as the exclusion restriction. The standard maximum likelihood (ML) estimate is shown, as well as the bias corrected estimate which is the mean of the posterior probability distribution. Also shown is the 95% bootstrap confidence interval for the bias corrected estimate, based on 1,000 replications. The bootstrap confidence interval is calculated using the empirical distribution of bootstrap estimates. Details of the statistical procedure are outlined in the appendix (see Additional file 1). Source: DHS Zambia 2007 (men).

**Figure 2:**
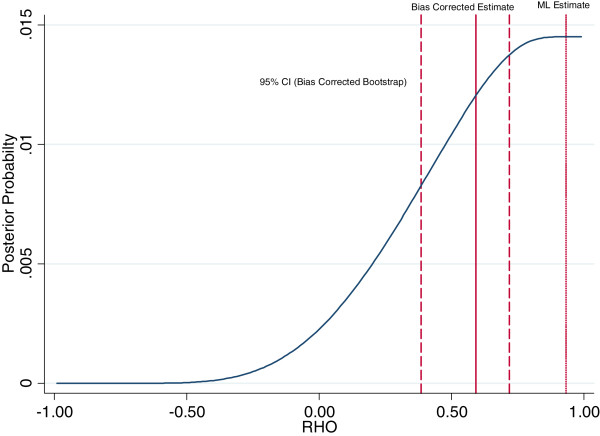
**Posterior probability distribution for the correlation between HIV Testing and HIV Status in Ghana 2003 (Men).** Graph shows the posterior probability distribution for the correlation between testing and HIV status *ρ* = *corr*(*u*_*ij*_, *ϵ*_*ij*_), calculated using a selection model with a flat prior probability distribution over the interval [-1,1], and interviewer random effects as the exclusion restriction. The standard maximum likelihood (ML) estimate is shown, as well as the bias corrected estimate which is the mean of the posterior probability distribution. Also shown is the 95% bootstrap confidence interval for the bias corrected estimate, based on 1,000 replications. The bootstrap confidence interval is calculated using the empirical distribution of bootstrap estimates. Details of the statistical procedure are outlined in the appendix (see Additional file 1). The fact that the maximum likelihood estimate lies outside the bootstrap confidence interval for the bias corrected estimate reflects the fact that the posterior distribution has a long left hand tail which is not accounted for by the standard maximum likelihood estimator, and that we use the empirical distribution of the bootstrap estimates to allow for asymmetry when calculating the confidence interval. Source: DHS Ghana 2003 (men).

In addition to specifying the interviewer parameters as a random effect, we also propose a bias correction procedure which improves on the maximum likelihood estimator by including information on all values of the correlation between the error terms in testing and HIV status which have non-zero probability. In contrast, the maximum likelihood estimate (by definition, the value with the highest posterior probability) ignores less likely values, even where their posterior probability is positive. To implement this procedure, we calculate the likelihood for each value of *ρ* from -1 to +1. Given a uniform (or flat) prior probability distribution, an appropriate transformation results in an approximation to a posterior density function, which allows us to calculate the expected value. We use this uniform prior probability distribution for the following reasons. First, there is little evidence to justify other prior probability distributions. In this context we lack information on whether a particular set of values for *ρ* (the correlation parameter of interest) should be preferred, and the use of a uniform prior probability distribution is common [35]. We have little evidence to expect any particular value for *ρ* to be more realistic than another. Second, from a practical perspective, the use of a uniform prior probability distribution makes it straightforward to implement our approach in standard software because we can obtain the posterior density function directly from the likelihood. We have also considered a fully Bayesian model for all parameters; however this approach would require additional prior probability distributions for all parameters and the implementation of a simulation procedure such as Markov Chain Monte Carlo [36]. Our methodology is designed to be easy to implement so that researchers can produce both prevalence estimates and valid confidence intervals given their survey data in a straightforward way. Finally, we note that as the sample size becomes large, we expect the choice of prior probability distribution to have a negligible effect on the resulting estimates [37]. However, as more reliable evidence becomes available on the relationship between testing and HIV status in different contexts, future research could consider adopting alternative approaches to specifying the prior probability distribution.This bias correction approach avoids the problem of boundary estimates, and is feasible to implement even when the likelihood function is monotonic. The difficulties associated with the maximum likelihood estimate, and the rationale for the bias correction, are illustrated in Figures 1 and 2, which demonstrate that when the likelihood is skewed, the most likely value for the correlation parameter and the expected value can differ substantially.

A major source of convergence failure in Heckman-type selection models (once the convergence problems caused by interviewer identity have been solved by the random effects approach) is in estimating the correlation parameter, *ρ*. The advantage of the bias correction approach is that we fix *ρ* at a particular value, and then estimate the corresponding log likelihood, so *ρ* does not have to be estimated in the model. Therefore, it should be possible to implement this method even when the fixed effects and random effects models fail. Full details of the statistical approach are provided in the appendix (see Additional file 1).

### Data

As the main goal of this paper is to introduce a new method for estimating HIV prevalence, we focus on two datasets from the Demographic and Health Surveys, Zambia (2007) and Ghana (2003), which include nationally representative HIV surveys of men aged 15–59. These data were chosen for two reasons. The first reason is that the results of our random effects model can be compared with the previous fixed effects approach which has already been applied to Zambia. The second reason is that the low HIV prevalence in Ghana makes estimation potentially problematic. Using the standard fixed effects approach, we find the correlation between being HIV positive and testing in Ghana to be large and positive (close to a correlation of *ρ* = 1), so that the maximum likelihood estimate for HIV prevalence among those who refuse to test is close to zero. In addition, in scenarios where the correlation between HIV status and non-participation in testing is large in absolute value, the usual models tend to fail to converge [20, 26, 27], making implementation of standard selection models difficult.

The analysis samples consist of 7,134 men in Zambia, and 5,334 men in Ghana. Table 1 shows the numbers testing for HIV, the numbers who undertook a DHS interview but refused consent to test for HIV, and the numbers who did not undertake an individual interview and did not test (non-contacts). Overall, participation rates in HIV testing were 72% in Zambia and 80% in Ghana. In this paper, we focus on those who refuse to consent to test. In principle, rather than having two separate models – one for contact and one for consent conditional on contact, we should have a single sequential model since requesting a test only occurs after people are interviewed [18]. However, non-contact is relatively rare, and previous studies found little evidence of selection bias among those who were not interviewed in Zambia [15]. We therefore impute HIV prevalence for non-contacts based on the characteristics of the person as reported by the household respondent, and the assumption that their HIV status is missing at random conditional on these covariates.

**Table 1:** **Sample size for HIV prevalence estimation among Men in Zambia (2007) and Ghana (2003)**

	Zambia		Ghana	
	N	%	N	%
Observed HIV Status	5,163	72%	4,271	80%
Missing HIV Status (Consent Refused)	1,318	18%	743	14%
Missing HIV Status (No Contact)	653	9%	320	6%
Total	7,134	100%	5,334	100%

Source: Demographic and Health Surveys.

A summary of the datasets used in the analysis are available from the DHS for both Zambia (http://dhsprogram.com/pubs/pdf/FR211/FR211%5Brevised-05-12-2009%5D.pdf) and Ghana (http://dhsprogram.com/pubs/pdf/FR152/FR152.pdf). We follow the approach adopted in previous papers used to prepare the DHS data for analysis of missing data in HIV surveys, the Stata code for which is publicly available from http://hdl.handle.net/1902.1/17657 [2, 15]. Table A1 in the appendix lists the variables used in the analysis and a description of their source in the data (see Additional file 1). Table A2 in the appendix presents information on the median number of interviewees, median consent rates, and median HIV prevalence rates by interviewer. In Zambia, 89 interviewers attempted to obtain consent to an HIV test. The median interviewer conducted 25 interviews, obtained a consent rate of 84%, and of those who consented to test for the median interviewer, 10% were HIV positive. In Ghana, 55 interviewers attempted to obtain consent to an HIV test. The median interviewer conducted 114 interviews, obtained a consent rate of 87%, and of those who consented to test for the median interviewer, 1% were HIV positive. A potential limitation of the random effects model in general is that when there are small number of groups, non-convergence can occur. However, in our data (and in many similar ones, such as most DHS), we have a relatively large number of interviewers (89 in Zambia and 55 in Ghana).

Interviewers who obtain 100% consent rates may be particularly informative, because there is no selection bias for these interviewers. Unfortunately, in our datasets (Zambia and Ghana), very few interviewers obtain 100% consent rates. Likewise, there are almost no interviewers with 0% consent rates. Of the 89 interviewers in total who attempted to obtain consent in Zambia, there were 21 interviewers with 100% consent rates and one with 0% consent; however most of these interviewers conducted a single interview, and all of them conducted less than 10 interviews each. Less than 2% of respondents were interviewed by one of these interviewers. Similarly, for Ghana relatively few respondents were interviewed by an interviewer who obtained 100% or 0% consent rates. These distributions highlight the major advantage of our random effects model, which makes dealing with these interviewers with few interviews much easier from a technical perspective than in the fixed effects framework: we are able to estimate an interviewer effect for all interviewers, even for those who conducted only a single interview. However, because of their limited number, it is difficult to obtain much information from these interviewers in our data, and they are not very informative for estimating HIV prevalence in this case. Further details on the interviewers are provided in Figures A1 – A4 in the appendix (see Additional file 1).

### Ethics approval

This study obtained an exemption from full ethics committee review at Harvard University. All data are publicly available and were analyzed anonymously.

## Results and discussion

We estimate HIV prevalence using three different selection models for Zambia and Ghana. First, we replicate the standard interviewer fixed effects method. Second, we implement the interviewer random effects approach without bias correction, and third, we use interviewer random effects with the bias correction. In addition, we compare these results to an imputation based model and analysis of cases with a valid HIV test (observations with complete data, where we exclude respondents with missing values).

Table 2 shows results for Zambia. The imputation model generates an estimate of 12% for population HIV prevalence, which is substantially smaller than the 20% estimate from the fixed effects model. The random effects and bias correction models give estimates that fall in between these two estimates (16%). Comparing the bootstrap and analytic standard errors highlights how the precision of these estimates is greatly overstated when not accounting for uncertainty in the estimation of *ρ*. Figure 1 shows the posterior probability distribution for the correlation parameter in Zambia, along with the maximum likelihood estimate, the bias corrected estimate based on the mean of the posterior probability distribution, and associated bootstrap confidence intervals. In this case, the posterior probability distribution is close to being symmetric, and the mode (the maximum likelihood estimate) is close to the mean.

**Table 2:** **Estimates of HIV prevalence among Men in Zambia (2007)**

Model	HIV prevalence	Analytic 95% CI		Bootstrap 95% CI	
All Men - Fixed Effects Selection Model	20.1%	19.0%	21.3%		
All Men - Random Effects Selection Model	16.3%	15.3%	17.3%	11.0%	18.4%
All Men – Random Effects Bias Correction Selection Model	15.5%	14.5%	16.5%	10.2%	17.9%
Men with Valid HIV Tests	12.1%	11.0%	13.3%		
Men with No Contact - Imputation Model	15.3%	14.2%	16.3%		
All Men - Imputation Model	12.3%	11.4%	13.2%		

In the Heckman-type selection models (rows 1-3), consent to test and HIV status are jointly estimated using a bivariate probit with the following covariates: education, household wealth quintile, type of location, marital status, had a sexually transmitted disease, age at first intercourse, had high risk sex, number of partners, condom use, would care for an HIV-infected relative, knows someone who died of AIDS, previously tested for HIV, smokes, drinks alcohol, language, age group, region, ethnicity and religion. The selection variable which predicts consent but not HIV status is interviewer identity. Full parameter estimates are presented in tables A4-A6 in the appendix (Additional file 1). Analytic standard errors are shown for the fixed effects and random effects models, with bootstrap errors for random effects and random effects bias correction models based on 1,000 replications. Our cluster bootstrap takes account of survey design by drawing a fixed number of clusters (the same as in the original data) from each stratum in each sample. Results from an imputation model are also shown in rows 5–6, along with estimates only using those without missing data (respondents with a valid HIV test). HIV prevalence estimates are weighted. Source: DHS Zambia 2007 (men).

In Ghana, the maximum likelihood estimator is difficult to implement because of low HIV prevalence. In addition, the relatively small sample size and low HIV prevalence may induce bias. Figure 2 illustrates the estimated posterior probability distribution for *ρ*. The concentrated likelihood function is approximately monotonic, resulting in maximum likelihood estimates which are close to the boundary of the parameter space, *ρ* = 1, and the implausible prediction that almost all those who decline to test are HIV negative. The maximum likelihood estimate places zero weight on all the values in the left hand tail of the posterior distribution in Figure 2 as they are less probable than the most likely value. To obtain our bias corrected estimate, we integrate over the posterior probability density function to account for all values of *ρ* which have positive probability. Under the assumption that the prior probability distribution is correct, this will result in an unbiased estimate for *ρ*. We obtain a value of around 0.6, indicating that individuals with HIV are more likely to consent to test in Ghana.

Table 3 reports our population prevalence estimates for Ghana. Relatively narrow confidence intervals reflect the low HIV prevalence in Ghana, and the lack of potential variation in HIV status in bootstrap replicate samples. For the selection models, we find HIV prevalence of 1.4%, compared to 1.6% in the case of the imputation model. Our population prevalence estimates are therefore slightly lower than those obtained from imputation methods. The positive selection we find in Ghana has also been found in other contexts, although it is in contrast to the negative selection found in Zambia [15]. Differences in selection mechanisms may reflect differences in HIV prevalence and other country-specific factors. For example, being HIV positive is unlikely to be a common reason for non-consent in a country with very low HIV prevalence. However, the differences in prevalence estimates are small in absolute terms, and therefore we conclude that there is little evidence that prevalence estimates are affected by selection bias among Ghanaian men. Tables showing parameter estimates from all models are presented in the appendix (see Additional file 1).

**Table 3:** **Estimates of HIV Prevalence among Men in Ghana (2003)**

Model	HIV prevalence	Analytic 95% CI		Bootstrap 95% CI	
All Men - Fixed Effects Selection Model	1.4%	1.1%	1.7%		
All Men - Random Effects Selection Model	1.4%	1.1%	1.7%	1.2%	1.6%
All Men - Random Effects Bias Correction Selection Model	1.4%	1.1%	1.7%	1.2%	1.6%
Men with Non-Missing Data (Valid HIV Test)	1.6%	1.2%	2.0%		
Men with No Contact - Imputation Model	1.6%	1.4%	1.8%		
All Men - Imputation Model	1.6%	1.3%	2.0%		

Consent to test and HIV status are jointly estimated using a bivariate probit with the following covariates: education, wealth quintile, marital status, had a sexually transmitted disease, age at first intercourse, had high risk sex, number of partners, condom use, would care for an HIV-infected relative, knows someone who died of AIDS, previously tested for HIV, smokes, language, age group, region, ethnicity and religion. The selection variable which predicts consent but not HIV status is interviewer identity. Full parameter estimates are presented in tables A8-A10 in the appendix (see Additional file 1). Analytic standard errors are shown for the fixed effects and random effects models, with bootstrap errors for random effects and random effects bias correction models based on 1,000 replications. Our cluster bootstrap takes account of survey design by drawing a fixed number of clusters (the same as in the original data) from each stratum in each sample. Results from an imputation model are also shown in rows 5–6, along with estimates only using those without missing data (respondents with a valid HIV test). HIV prevalence estimates are weighted. Source: DHS Ghana 2003 (men).

## Conclusions

This paper confirms that non-participation in HIV testing may be an important source of bias in HIV prevalence estimation that does not correct for non-ignorable missing data. We introduce a random effects approach for Heckman-type selection models, which improves on previous fixed effects approaches by allowing us to estimate interviewer effects for all interviewers, even for interviewers with few interviewees. This approach makes it possible to use bootstrapping to calculate confidence intervals that account for the fact that the relationship between selection and HIV status is uncertain. We also introduce a bias correction model which facilitates estimation of the correlation between consent to test and HIV status when HIV prevalence or non-participation is very high or very low, and when the usual maximum likelihood model fails to converge. In Zambia, we find that men with HIV are less likely to consent to an HIV test. For Ghana, we find little evidence that conventional methods understate HIV prevalence.

An important result from our empirical analysis is that the corrected confidence intervals around the HIV prevalence point estimate can be very wide. These wide confidence intervals accurately reflect the fact that it is difficult to correct statistically for the bias that may occur when many people refuse to test for HIV. As long as consent rates are low, uncertainty in HIV prevalence estimation will likely remain high. It is important not to understate this uncertainty, and our approach provides a practical solution to account for both sampling and parameter uncertainty in the estimation of HIV prevalence confidence intervals when using Heckman-type selection models to remove selection bias.

The goal of this paper is to address key limitations of standard Heckman-type selection models when a valid selection variable – in this case, interviewer identity – is available. While it is plausible that interviewer allocation is only affected by survey design and not associated with respondents’ HIV status, this claim is not possible to prove conclusively without additional data, because the HIV status of those who do not participate in testing is not observed. As the resulting HIV prevalence prediction relates to a population for whom we generally never get to observe true HIV status, it is important to independently validate the model. We are therefore working to obtain objective data in the form of mortality or antiretroviral treatment records with which we can do so [38].

A drawback of the random effects procedure is that it is performed in two steps: first, the interviewer random effects are estimated from a probit model, and then the interviewer random effects are included in the selection model as the selection variable. This additional step of requiring the interviewer effects to be estimated could introduce measurement error into the model. This may be reflected in the slightly lower HIV prevalence estimates we find for the random effects model in Zambia compared to the fixed effects model. Developing the methodology to incorporate the interviewer random effects directly into bivariate probit selection models, or other approaches to eliminate this potential attenuation bias, would be a useful advance. Another general drawback of selection models for binary outcomes is that they require a parametric assumption. We are pursuing alternative methods in order to determine the robustness of these results to violation of the assumption that the error terms in the selection and HIV status equations are distributed as bivariate normal. These limitations, coupled with the wide confidence intervals we find in the empirical analyses, suggests that despite methodological improvements to remove selection biases from HIV prevalence estimates, it is critical to increase consent rates in HIV surveys. In countries with high non-participation in HIV surveys, interventions to increase consent, such as financial incentives, should be considered for routine implementation.

## Endnotes

^a^Further details of the testing procedure are available online at http://dhsprogram.com/pubs/pdf/OD61/OD61.pdf.

^b^Which can be thought of as reflecting propensity to be infected.

^c^The identity of the interviewer who sought to contact eligible participants is also typically available in the DHS as an anonymized code.

^d^Therefore, although we mainly refer to declining consent to test for HIV in this paper, it is straightforward to apply this approach to missing data which arises through other mechanisms which result in non-participation.

^e^Because there is no within-interviewer variation in consent for these interviewers, and these interviewer parameters perfectly predict consent to test for their interviewees.

^f^Practical solutions to the boundary problem in bivariate probit models have been proposed, but these do not necessarily solve the difficulty of being left with implausibly large correlation coefficients [27, 39].

## Electronic supplementary material

Additional file 1: **Further description of the statistical approach and additional results.** (PDF 638 KB)

## Abbreviations

HIV: Human immunodeficiency virus
DHS: Demographic and Health Surveys
MAR: Missing at random
ML: Maximum likelihood
CI: Confidence interval
PSU: Primary sampling unit.
