# Supplementary material for: Using interviewer random effects to remove selection bias from HIV prevalence estimates
Source: BMC Med Res Methodol. 2015 Feb 5;15:8. doi: 10.1186/1471-2288-15-8 (PMC4429465; doi:10.1186/1471-2288-15-8)
Supplement: Supplementary file 1 — Additional file 1: Further description of the statistical approach and additional results. (PDF 638 KB) [file 12874_2014_1178_MOESM1_ESM.pdf]

Appendix to: Using Interviewer Random Effects to Remove Selection Bias from HIV Prevalence Estimates

Mark E. McGovern*† Till Bärnighausen‡ Joshua A. Salomon§ David Canning¶

Additional File 1 - Further description of the statistical approach and additional results

A1 Statistical Approach

A1.1 Standard Heckman-Type Selection Model for Estimating HIV Prevalence

As outlined in the main text, our Heckman-type selection model adopts a bivariate probit approach [1, 2], where we model consent to test for HIV for person i with interviewer j as the observed outcome arising from a latent variable that may be interpreted as the propensity to consent to testing [3]:

$$s_{ij}^{*} = {x_{ij}}'\beta_s + {z_j}'\gamma_s + u_{ij} \tag{A1}$$

$$s_{ij} = 1 \text{ if } s_{ij}^{*} > 0, s_{ij} = 0 \text{ otherwise}$$

where s_{ij} is a binary indicator for agreeing to test, x_{ij} are observed characteristics, z_j are interviewer effects, u_{ij} is random error, and s_{ij}^{*} is an unobserved latent variable. β_s and γ_s are the associated vectors of parameters.

*Corresponding author. Address: 9 Bow Street, Cambridge, MA 02138, USA.

†Harvard Center for Population and Development Studies and Department of Global Health and Population, Harvard T.H. Chan School of Public Health. Email: mcgovern@hsph.harvard.edu.

‡Department of Global Health and Population, Harvard T.H. Chan School of Public Health, and Wellcome Trust Africa Centre for Health and Population Studies, South Africa. Email: tbaernig@hsph.harvard.edu.

§Department of Global Health and Population, Harvard T.H. Chan School of Public Health. Email: jsalomon@hsph.harvard.edu.

¶Harvard Center for Population and Development Studies and Department of Global Health and Population, Harvard T.H. Chan School of Public Health. Email: dcanning@hsph.harvard.edu.

The equation for the HIV status of individual i with interviewer j is:

$$h^*_{ij} = x_{ij}{'}\beta_h + \epsilon_{ij} \tag{A2}$$

$$h_{ij} = 1 \text{ if } h^*_{ij} > 0, h_{ij} = 0 \text{ otherwise}$$

$$h_{ij} \text{ observed only if } s_{ij} = 1$$

where h^*_{ij} is again a latent variable, which can be thought of as reflecting propensity to be infected, and ϵ_{ij} is an error term. We assume (u_{ij}, ϵ_{ij}) are jointly distributed as bivariate normal, each with mean zero, variance 1, and correlation parameter $\rho = corr(u_{ij}, \epsilon_{ij})$.

The inclusion of the interviewer effects, z_j, which are assumed to affect consent to testing, but not HIV status, is crucial to the model. Without variables in the selection equation that are excluded from the HIV status equation, the model is only identified by non-linearities, and does not provide robust estimates [4].

Even with a suitable exclusion restriction, there are a number of technical problems associated with estimating the model. The first concerns the interviewer effects in z_j, which typically take the form of a series of binary indicator variables, essentially an interviewer fixed effect [3]. Estimation of these interviewer fixed effects may be difficult because, for interviewers who have only successes or failures in obtaining consent, the parameter is not identified. For example, if an interviewer always obtains consent, we know that the coefficient on the indicator variable for that interviewer in the consent equation is large and positive. However, the coefficient is not identified, as there is no variation in consent for that interviewer. The coefficient for that interviewer could be arbitrarily large, as any very large positive interviewer effect above some threshold will perfectly predict success, and therefore we cannot distinguish between different effect sizes for interviewers who obtain 100% consent. In addition, we control for the region and language of the interviewee. However, in the DHS interviewers usually work in a small number of regions, and an effort is often made to match them with interviewees by language. This may create collinearity between the interviewer effect and the region and language indicator variables in the equation, making it difficult or even impossible to estimate the interviewer effects. The approach adopted by Bärnighausen and colleagues was to limit estimation of individual interviewer effects to those interviewers with more than 50 interviews, and not to use the interviewer effects of those interviewers who had conducted more than 50 interviews when including them lead to identification problems [3].[1] Instead, interviewers with less than 50 interviewers, or interviewers whose inclusion caused identification problems, were grouped together as a baseline group with an assumed common interviewer effect.

A conceptual difficulty is that grouping all of the interviewers whose individual interviewer effects are not identified into a baseline group may not be a convincing strategy, because we could be pooling interviewers who always have consent with those who never achieve consent, and assuming that they have the same average effectiveness in obtaining consent. Thus, we do not exploit the fact that the HIV tests made by interviewers who always obtain consent are the most informative

[1]The identification problem is usually manifested in practice by the model failing to converge, technically the unidentified parameters have estimated standard errors that are large and increasing at every iteration – the limit is an unbounded standard error on the unidentified parameter.

since they do not suffer from selection bias. As we discuss in the main text, two other limitations with the fixed effect approach are potential small sample bias, and failure of the bootstrap method for calculating standard errors.

A1.2 Selection Model Using Random Effects

Our approach to these problems is to assume that the unobserved interviewer effects reflect some underlying parametric distribution which describes interviewer effectiveness. If interviewers were randomly assigned to survey households we could simply assume that each interviewer was a random draw from the pool of interviewers. However, systematic matching of interviewers to subjects, particularly by region and language, can mean that there may be a correlation between interviewer success rates and who they are matched with. Therefore, we write the interviewer effect as [5, 6]:

$$z_j = \bar{x}_j' \delta + v_j \tag{A3}$$

$$\bar{x}_j = \sum_{i}^{n_j} \frac{x_{ij}}{n_j}, v_j \sim N(0, \sigma_z^2)$$

Where $\bar{x}_j$ represents the average characteristics of the n_j people that interviewer j interviews. Excluding these controls could potentially invalidate the exclusion restriction of the model. In order to be asymptotically unbiased and consistent, a requirement of random effects models is that there is no correlation between the random effects, v_j, and the explanatory variables at the individual level, x_{ij} [5, 6]. In order for this to hold, we must assume that, controlling for these observable averages, there is no remaining correlation between the error term in equation (A3) and the individual level variables in the model. In particular, we assume that interviewers are not systematically assigned to groups of survey participants based on unobservable characteristics, but only on the observable characteristics measured by the survey. As these $\bar{x}_j$ likely depend only on survey design, they should not enter the HIV status equation.[2]

This assumption of interviewer random effects gives us the selection equation:

$$s_{ij}^* = {x_{ij}}' \beta_s + \bar{x}_j' \delta + v_j + u_{ij}$$

$$s_{ij} = 1 \text{ if } s_{ij}^* > 0, s_{ij} = 0 \text{ otherwise} \tag{A4}$$

In principle, we could estimate the system given by equations (A2) and (A4) by maximum likelihood. However, this is difficult as we have a selection equation which has a random effect, requiring numerical integration, inside a bivariate probit model.

There is, however, a simple consistent estimator. Dubin and Rivers show that the bivariate probit model with selection can be estimated by first finding a consistent estimate of the parameters of the selection model, ignoring the covariance of the error terms, and then estimating the parameters

[2] If they are included in the HIV status equation they have little effect on our results; they simply reduce the efficiency of estimation.

of the full model by maximum likelihood, holding the selection equation parameters at their first stage estimates [2]. The procedure is as follows. We first estimate the interviewer effects from the selection equation only (stage one), then include these constructed parameters as our exclusion restriction in a Heckman-type selection model (stage two). This two-stage approach is consistent, though not fully efficient, and the second stage does not produce the correct analytic standard errors since the interviewer effects are estimated in the first stage, but treated as exogenous variables in the second stage. Murphy and Topel discuss the consequences for confidence intervals in models which use data that are estimated from a first stage [7].

We can implement this approach by first estimating the interviewer effect as shown in equation (A3). Equation (A4), the selection equation, is then run using the predicted interviewer effect ($z_j = \bar{x}'_j\delta + v_j$) as the exclusion restriction. Assuming random effects avoids the estimation problems of the fixed effects approach; because we are able to estimate an interviewer effect for all interviewers we can implement a bootstrap procedure to adjust our standard errors for the fact that the relationship between consent and HIV status is uncertain and needs to be estimated. The assumption that the interviewer effects are normally distributed random effects around $\bar{x}'_j\delta$ also means we have a smaller set of parameters to estimate than in the fixed effects model.

Conceptually, we could estimate the interviewer random effects using a multilevel regression for consent, with level one being the individual and level two being the interviewers. However, a simpler method of constructing consistent estimates of the interviewer random effects is to implement a probit model and compute the predicted random effect, $\hat{v}_j$, as the average of the error term for each interviewer.[3] We use this probit model to compute the estimated interviewer effect, $\hat{z}_j = \bar{x}'_j\hat{\delta} + \hat{v}_j$, where $\hat{\delta}$ is the vector of estimated coefficients on the interviewer averages from equation (A3), and $\hat{v}_j$ is the predicted random effect from equation (A3).[4] Having obtained the estimates $\hat{z}_j$, we can then estimate the full bivariate probit model in equations (A1) and (A2) using this estimated interviewer effect as the selection variable that affects consent but does not appear in the HIV status equation.

Since the first stage is a consistent estimator of the interviewer effect, the two-stage procedure will be consistent. We can address the problem of incorrect standard errors by bootstrapping over the whole two-stage procedure.

A1.3 Bias Correction

While the random effects approach solves the identification problems associated with the interviewer parameters and allows bootstrapping, a second problem remains. In small samples, particularly when consent to test is very rare (or very common), or the HIV rate is very low (or very high), it is difficult to estimate the correlation between consent to test and HIV status [8], and the maximum likelihood estimates in the context of bivariate probit models can be biased [9]. Also, the model may fail to converge, or may produce a result of $\rho = \pm 1$, on the boundary of the possible parameter space, in which case the assumptions required for asymptotic normality of the maximum likelihood

[3] Estimating this first stage as a random effects model is computationally intensive and produces almost identical results to the simple probit. The simple probit produces consistent estimates and this is all this is required for the second stage.

[4] Our results are robust to just using $\hat{v}_j$ alone as the interviewer effect rather than the full estimate $\bar{x}'_j\hat{\delta} + \hat{v}_j$

estimator are violated, and standard inference, including bootstrapping, is invalid [10, 11]. Such a result also has the implication that, in terms of predicted probabilities, everyone who fails to test is either HIV positive with certainty ($\rho = +1$), or HIV negative with certainty ($\rho = -1$), which seems implausible. In general, maximum likelihood often does not have desirable finite sample properties [12, 13], as the estimate is the most likely value (in terms of posterior probability), which gives zero weight to values with lower posterior probabilities, even when those probabilities are positive.

We wish to construct an estimate of ρ that is consistent and also corrects for this small sample bias. Take a data set x^* and a parameter vector θ^*. The likelihood of θ^* is simply the probability of the data given these parameters:

$$L(\theta^*) = P(x^*|\theta^*) \tag{A5}$$

In the usual maximum likelihood framework, we are not generally concerned with the likelihood of the observed data given the parameters, we are more interested in the probability of the parameters given the observed data. However, by Bayes rule:

$$P(x^*|\theta^*) = \frac{P(x^*|\theta^*)P(\theta^*)}{P(x^*)} \tag{A6}$$

Where $P(\theta^*)$ is our prior probability distribution on the parameters. If we have a flat prior probability distribution over the interval [-1, 1], so that $P(\theta^*)$ is the same for every θ^*, we have $P(\theta^*|x^*) \propto P(x^*|\theta^*) = L(\theta^*)$, and the maximum likelihood estimate of θ^* is also the estimate that has the highest posterior probability given the data. While this estimate is the most likely parameter value given the data, we can construct an alternative estimator as:

$$\hat{\theta^*} = E(\theta^*|x^*) = \int_{-\infty}^{+\infty} \theta^* P(\theta^*|x^*) d\theta^* \tag{A7}$$

If the prior probability distribution is flat, we have $P(\theta^*|x^*) \propto L(\theta^*)$, the posterior probability is proportional to the likelihood, and we can write our estimator as:

$$\hat{\theta^*} = E(\theta^*|x^*) = \int_{-1}^{+1} \theta^* k L(\theta^*) d\theta^* \tag{A8}$$

Where k is a normalization factor so that the integral of the likelihood over the parameter space is one, and $\theta^* k L(\theta^*)$ can be interpreted as an approximation to a probability density function.

The standard maximum likelihood approach chooses the most likely value of θ^*, while our approach gives us an average value of θ^*, where we average over different models weighted by the probability of the model being correct. This gives consistent estimates (the likelihood function asymptotically puts zero weight on incorrect parameters) and is an unbiased estimator by construction under the assumption that the prior probability distribution is correct. This approach is implemented by calculating the likelihood for each value of ρ, and then taking the weighted average of ρ, where the weights are the likelihood values (transformed so that these values integrate to 1).

In principle, we could construct these estimates for all the parameters in the model. However, in practice the maximum likelihood estimates for most of the parameters in the model are well determined,[5] and it is only the correlation parameter ρ that poses problems. We therefore use a profile (or concentrated) likelihood for ρ (see Appendix 3 for details). The profile likelihood can be used to substitute for the full likelihood when there are other parameters in the model which are not of direct interest [14]. We have verified that estimates for these other parameters are very stable across all models. Therefore, once ρ is estimated we find the maximum likelihood values of the other parameters given this value of ρ.

We report the maximum likelihood estimates and our new bias correction estimates together with bootstrapped standard errors and confidence intervals. Our cluster bootstrapping takes account of the stratification and cluster sampling procedure of the survey design [15]. All our HIV prevalence estimates are weighted.

[5] Once we have accounted for the difficulties associated with the interviewer parameters using the random effects approach.

A2 Additional Results for Model Parameters

We begin by estimating the interviewer random effects as the average error term (for each interviewer) from a probit model for consent to test for HIV, where we include a standard set of covariates along with the mean of these variables for each interviewer to capture the effects of non-random allocation of interviewers to participants as shown in equation (A4). Descriptive statistics for interviewers in Zambia and Ghana are shown in Table A1. Table A2 lists the variables used as predictors in the bivariate probit model, and their source in the data. The Stata code for preparing the data is publicly available from http://hdl.handle.net/1902.1/17657 [3, 16]. The distribution of the number of interviewees by interviewer and their consent rates is shown in figures A1-A4.

Table A3 presents results from the probit model for consent to test for HIV in Zambia. This table is a single regression for HIV consent where the first column shows the marginal effects for the individual level variables (x_{ij}), while the corresponding marginal effects for the interviewer averages ($\bar{x}_j$) are shown in the second column.[6] At the individual level, the following variables are associated with consent to test: education, location, prior sexually transmitted disease (STDs), age at first intercourse, number of partners, willingness to care for a HIV positive relative, knowing an AIDS victim, and being a smoker. For example, an extra year of education is associated with an increase in the probability of consenting to an HIV test by roughly 0.5 percentage points. Apart from years of education, which is the mean years of education for that interviewer's interviewees, all interviewer averages are measured as the proportion of interviewees in that category. Many of the interviewer average measures are significant in predicting consent. For example, in table A3 if the interviewee speaks Lozi the probability of consent to testing increases, but the association is not statistically significant. However, interviewers who conducted more interviews with Lozi speakers had higher consent rates (indicated by the positive and statistically significant coefficient for the proportion of interviews with Lozi speakers in table A3). These interviewers are likely to be Lozi speakers themselves, and these results therefore suggest that the Lozi speaking interviewers may have been better than average at obtaining consent.

Table A4 presents marginal effects for the Heckman-type selection model for consent and HIV status for Zambia. The first two columns give results for the maximum likelihood fixed effects approach. The middle two columns give results for our maximum likelihood random effects approach, and the final two columns give the results for the random effects bias correction model. For this third model, we estimate the likelihood on a grid of values of ρ (we use values between -1 and $+1$ at intervals of 0.01). We than calculate the likelihood of the model and posterior probability of each value of ρ, and then find the expected value of ρ based on this probability.[7] The coefficients reported in table A4 are calculated with this value of ρ imposed. Coefficient estimates across the three models are very similar.

Chiburis and colleagues recommend the use of the bootstrap for inference in the context of recursive bivariate probit models to correct for poor coverage of analytic standard errors [9]. We find that the bootstrap confidence interval for the random effects model is almost 10 times as wide as the analytic standard errors for the fixed effects model. We use 1,000 iterations to calculate

[6] Marginal effects show the expected change in the probability of a positive outcome for a unit change in that covariate, evaluated at the mean values of the covariates.

[7] The posterior probability is calculated by applying a constant to the likelihood for each value of ρ such that these transformed likelihoods integrate to 1.

these standard errors, and the corresponding 95% bootstrap confidence interval is calculated using the appropriate centiles from the empirical distribution of the bootstrap estimates. This approach is more appropriate than normal-based approximations when the distribution of the parameter of interest is skewed.

In table A5, we present the estimated correlation coefficient for Zambia from the different models. The negative values estimated indicate that those who refuse to test are more likely to be HIV-positive. The maximum likelihood random effects approach yields a ρ of around -0.50, which is somewhat lower than the -0.75 obtained from the fixed effects estimator. The random effects bias corrected estimate is slightly smaller again at -0.44. Table A6 presents estimates of HIV prevalence among those who refused to consent to an HIV test, again comparing the fixed effect, random effect and random effects bias corrected methods. As with the correlation coefficient, the estimates from the random effects model are lower than from the fixed effects model (32% v 52%). The corresponding estimate from the imputation model is 12%. Table A7 presents marginal effects for the consent model in Ghana, and tables A8-A10 give the parameter results of the three Heckman-type selection models.

A3 The concentrated (profile) likelihood function

The likelihood of the parameters (β_s, β_h, ρ), given the full data set (y, x, z) is:

$$L(\beta_s, \beta_h, \rho) = P(y, x, z|\beta_s, \beta_h, \rho) \tag{A9}$$

For a given ρ, we can concentrate the likelihood function by setting the other parameters at their maximum likelihood values given ρ:

$$L_c(\rho) = P(y, x, z|\hat{\beta}_s(\rho), \hat{\beta}_h(\rho), \rho) \approx P(y, x, z|\rho) \tag{A10}$$

In large samples, the approximation to $P(y, x, z|\rho)$ will become exact as the maximum likelihood estimates of the other parameters are consistent. Using the concentrated maximum likelihood, the problem is reduced to a one parameter model and we can carry out our small sample correction with a prior probability distribution over ρ alone.

A4 Additional Tables and Figures

Table A1: Descriptive Statistics by Interviewer

	Number of Interviewers	Number of Interviews by Interviewer	Consent by Interviewer	HIV Prevalence By Interviewer
		Median (Interquartile Range)		
Zambia	89	25 (4 – 132)	84% (72% – 93%)	10% (0% – 16%)
Ghana	55	114 (5 – 152)	87% (81% – 94%)	1% (0% – 2%)

Note to table A1: For each interviewer, their consent rate is calculated as the number of respondents from whom consent to test for HIV was obtained by the interviewer, divided by the number of respondents from whom consent to test for HIV was sought by the interviewer. For each interviewer, their HIV prevalence rate is calculated as the number of HIV positive respondents among those who consented to test for that interviewer, divided by the number of respondents who consented to test for that interviewer.

Table A2: DHS Variable Description

Variable	DHS Variable Name (Zambia 2007)	Note
**Demographic Characteristics**		
Age Category	v013	5 year age bands
Years of Education	v133	Education in single years
Marital Status	v501	Never/previously/currently married
Ethnicity	v131	Categorical
Language	smlangi	Language of interview
Religion	v130	Categorical
**Sex and Behavior**		
Would Respondent Care for HIV Positive Relative?	v778	Binary Indicator
Does Respondent Know Anyone Who Died of AIDS?	v775	Binary Indicator
Ever had an HIV Test	v781	Binary Indicator
Smoker	v463a, v463b, v463c	Binary Indicator
Alcohol Drinker	s1012a	Binary Indicator
Ever Had STD	v763a, v763b, v763c	Binary Indicator
Age at First Intercourse	v525	Never, <age 15, >age 15
Had High Risk Sex	v766a	Binary, sex with someone other than partner
Used Condom Last Intercourse	v761	Binary Indicator
Number of Partners Last 12 Months	v766a, v766b	Continuous
**Household Characteristics**		
Wealth Quintile	mv190	Categorical
Type of Location	hv025	Large City/Small City/Town/Countryside
Region	v101	Categorical
**HIV Testing**		
Consented to Test for HIV	ha63	Binary Indicator
Valid HIV Test Result	hb63	Binary Indicator
**Survey Characteristics**		
Interviewer Identity	v028	Anonymised ID Code
Primary Sampling Unit	v001	PSU Cluster
Household Weight	hv005	Nationally representative weight

Table A3: Marginal Effects from a Probit Model for HIV Test Consent for Zambian Men

	HIV Test Consent	
Variable	Individual Level Variables	Interviewer Level Averages
Years of Education	0.00475**	-0.0001
	(0.002)	(0.000)
**Wealth Category**		
Poorest	-0.00731	-0.0086***
	(0.031)	(0.002)
Poorer	-0.02721	-0.0046
	(0.031)	(0.003)
Middle	-0.03732	-0.0059
	(0.027)	(0.004)
Richer	-0.00842	-0.0109***
	(0.019)	(0.004)
**Location**		
Small city	0.12471***	0.0039
	(0.037)	(0.004)
Town	0.14458***	0.0036
	(0.044)	(0.004)
Countryside	0.20348***	0.0008
	(0.062)	(0.003)
**Marital Status**		
Never Married	0.01165	0.0036
	(0.028)	(0.003)
Currently Married	0.03447	-0.0018
	(0.031)	(0.004)
Had STD	0.04105*	0.0036
	(0.022)	(0.003)
**Age at First Intercourse**		
15 or Younger	0.04986**	0.0043
	(0.020)	(0.003)
>15	0.03778*	0.0013
	(0.022)	(0.003)
**Had High Risk Sex**	0.05241**	-0.0038
	(0.025)	(0.004)
**Number of Partners**		
None	0.05095**	-0.0078**
	(0.025)	(0.004)
2+	0.01516	0.0021
	(0.021)	(0.003)
Used Condom Last Intercourse	-0.00033	-0.0005
	(0.016)	(0.002)

Table A3 – Continued on the Next Page

	HIV Test Consent	
Variable	Individual Level Variables	Interviewer Level Averages
Would Care for HIV Relative	0.05757**	0.003
	(0.024)	(0.003)
Knows Someone Who Died of AIDS	0.03795***	-0.0011
	(0.011)	(0.001)
Previously HIV Tested	0.00504	-0.0038
	(0.013)	(0.002)
Smoker	0.03644***	0.0041**
	(0.012)	(0.002)
Drinks Alcohol	0.01008	-0.0064***
	(0.012)	(0.002)
**Language**		
English	-0.04601	0.0079
	(0.062)	(0.008)
Bemba	-0.00834	0.0037
	(0.061)	(0.009)
Lozi	0.04688	0.0130**
	(0.056)	(0.007)
Nyanja	0.05083	0.0036
	(0.055)	(0.009)
Tonga	0.06172	0.006
	(0.062)	(0.008)
Observations	6,416	

Robust standard errors in parentheses
*** p <0.01, ** p <0.05, * p <0.1

Note to table A3: Age group, region, religion, and ethnicity are included in the regression but are not shown. Each column is for the same regression where HIV consent is regressed on the X variables and the corresponding average (in percent) for each interviewer. A probit model is used, and marginal effects (absolute change in the probability of a positive outcome) are shown. In some cases standard errors are very small, but not zero as appears in the table due to rounding. Interviewer averages of categorical variables are a proportion ranging from 0 to 1. Standard errors account for clustering at the PSU level.

Table A4: Marginal Effects for Heckman-type Selection Models (Men Zambia 2007)

	Maximum Likelihood Fixed Effects		Maximum Likelihood Random Effects		Bias Correction Random Effects	
Variables	Consent Equation	HIV Equation	Consent Equation	HIV Equation	Consent Equation	HIV Equation
Interviewer Effect				0.2845***		0.2857***
				(0.028)		(0.028)
Years of Education	0.0046**	0.0012		0.0019	0.0047**	0.002
	(0.002)	(0.002)	(0.002)	(0.002)	(0.002)	(0.002)
**Wealth Category**						
Poorest	-0.0172	0.0486**	-0.0199	0.0463**	-0.0198	0.0451**
	(0.020)	(0.020)	(0.020)	(0.018)	(0.020)	(0.018)
Poorer	-0.0262	0.0528***	-0.0299	0.0479***	-0.0298	0.0465***
	(0.021)	(0.020)	(0.021)	(0.018)	(0.021)	(0.018)
Middle	0.0028	0.0645***	-0.0017	0.0630***	-0.0017	0.0620***
	(0.025)	(0.024)	(0.024)	(0.022)	(0.024)	(0.021)
Richer	0.0055	0.0503*	0.0072	0.0493*	0.0071	0.0485*
	(0.031)	(0.028)	(0.030)	(0.025)	(0.030)	(0.025)
**Location**						
Small City	0.1529**	-0.0589	0.1626**	-0.0351	0.1622**	-0.0308
	(0.067)	(0.045)	(0.064)	(0.035)	(0.064)	(0.032)
Town	0.1605***	-0.0764**	0.1649***	-0.0495*	0.1647***	-0.0449*
	(0.061)	(0.038)	(0.059)	(0.027)	(0.059)	(0.024)
Countryside	0.1911***	-0.1289***	0.1972***	-0.0956***	0.1967***	-0.0896***
	(0.063)	(0.039)	(0.059)	(0.028)	(0.059)	(0.024)
**Marital Status**						
Currently Married	0.0105	0.0787***	0.022	0.0791***	0.0221	0.0779***
	(0.029)	(0.030)	(0.028)	(0.027)	(0.028)	(0.027)
Formerly Married	-0.0146	0.2125***	-0.0093	0.1992***	-0.0096	0.1950***
	(0.028)	(0.026)	(0.028)	(0.025)	(0.028)	(0.023)
Had STD	0.0407	0.1079***	0.0437*	0.1073***	0.0437*	0.1064***
	(0.025)	(0.023)	(0.025)	(0.019)	(0.025)	(0.019)
**Age at First Intercourse**						
15 or Younger	0.0502**	-0.0422	0.0485**	-0.0254	0.0487**	-0.0228
	(0.022)	(0.035)	(0.021)	(0.031)	(0.021)	(0.030)
>15	0.0374*	-0.0475	0.0356*	-0.0334	0.0357*	-0.0312
	(0.022)	(0.034)	(0.021)	(0.030)	(0.021)	(0.030)
**Had High Risk Sex**	0.0531*	-0.0275	0.0546**	-0.0199	0.0544**	-0.0184
	(0.027)	(0.025)	(0.027)	(0.022)	(0.027)	(0.021)
**Number of Partners**						
1	-0.0433	-0.0202	-0.0520*	-0.0266	-0.0521*	-0.0272
	(0.027)	(0.029)	(0.027)	(0.026)	(0.027)	(0.025)
2+	-0.0259	0.0343	-0.0371	0.0293	-0.0371	0.0282
	(0.042)	(0.039)	(0.042)	(0.034)	(0.042)	(0.033)
Condom Last Intercourse	-0.0079	0.0629***	-0.0017	0.0586***	-0.0014	0.0575***
	(0.016)	(0.015)	(0.015)	(0.014)	(0.015)	(0.013)
Would Care for HIV Relative	0.0487**	0.017	0.0524***	0.0285	0.0525***	0.0298
	(0.020)	(0.033)	(0.020)	(0.030)	(0.020)	(0.029)
Know Someone Who Died of AIDS	0.0369***	-0.0144	0.0370***	-0.0081	0.0370***	-0.007
	(0.010)	(0.014)	(0.010)	(0.011)	(0.010)	(0.011)
Previously HIV Tested	0.0053	0.0340***	0.005	0.0333***	0.005	0.0328***
	(0.013)	(0.013)	(0.013)	(0.011)	(0.013)	(0.011)
Smoker	0.0371***	-0.0262*	0.0371***	-0.0197	0.0370***	-0.0183
	(0.013)	(0.016)	(0.013)	(0.013)	(0.013)	(0.013)
Drinks Alcohol	0.0097	0.0186	0.0106	0.0184*	0.0105	0.0182*
	(0.012)	(0.012)	(0.012)	(0.011)	(0.012)	(0.011)
**Language**						
English	0.0276	0.0028	0.035	0.0075	0.0351	0.0081
	(0.028)	(0.033)	(0.029)	(0.029)	(0.029)	(0.029)
Bemba	0.0953	0.0666	0.0916	0.0816*	0.0919	0.0826**
	(0.061)	(0.049)	(0.058)	(0.042)	(0.058)	(0.041)
Lozi	0.0980***	-0.0076	0.0967***	0.0079	0.0965***	0.0099
	(0.030)	(0.031)	(0.028)	(0.026)	(0.028)	(0.025)
Nyanja	0.1017**	-0.0177	0.1133***	0.0035	0.1132***	0.0066
	(0.046)	(0.043)	(0.036)	(0.035)	(0.036)	(0.034)
Tonga	0.0376	-0.0119	0.0453	-0.0132	0.0452	-0.0128
	(0.048)	(0.064)	(0.051)	(0.063)	(0.051)	(0.062)
Observations	6,416	6,416	6,416	6,416	6,416	6,416

Robust standard errors in parentheses
*** p <0.01, ** p <0.05, * p <0.1

Note to table A4: Controls for age group, region, ethnicity, and religion are included in each regression equation but not shown. The model is a bivariate probit for HIV status and consent to a HIV test. Data are for men who completed an interview. The first model uses interviewer fixed effects as the exclusion restriction. The coefficients from the interviewer effects are not shown in the table. The second model uses the random effects procedure where HIV consent is regressed on the X variables, along with the mean values for each interviewer (see table A3). The average error term for each interviewer is added to the predicted value of the interviewer means, and used as the exclusion restriction in the HIV regression. The final model is the random effects bias correction procedure using the same exclusion restriction. Coefficients are obtained by restricting the value of ρ to its random effects bias correction estimate and implementing the bivariate probit at that value. Marginal effects (absolute change in the probability of a positive outcome) are shown. Source: DHS Zambia 2007 (men). Standard errors account for clustering at the PSU level.

Table A5: Correlation Coefficient (ρ) Estimates for Men in Zambia

Model for Refused Consent	Parameter Value	Analytic 95% CI		Bootstrap 95% CI	
Fixed Effects Model	-0.75	-0.94	-0.22		
Random Effects Model	-0.50	-0.73	-0.18	-0.68	0.23
Random Effects Bias Correction Model	-0.44			-0.64	0.43

Note to table A5: The table shows the estimated correlation coefficient between consent and HIV status for the fixed effects, random effects and random effects bias correction models. Analytic standard errors are shown for the fixed effects and random effects models, with bootstrapped errors for random effects and random effects bias correction models using 1,000 replications. Source: DHS Zambia 2007 (men).

Table A6: HIV Prevalence (%) among Men who Refused to Test in Zambia

Model for Refused Consent	Parameter Value	Analytic 95% CI		Bootstrap 95% CI	
Fixed Effects Model	52.00%	49.90%	54.00%		
Random Effects Model	32.00%	30.30%	33.80%	6.30%	42.70%
Random Effects Bias Correction Model	28.60%	26.60%	29.80%	3.20%	39.80%
Imputation Model	11.70%	10.80%	12.60%		

Note to table A6: The table shows the estimated HIV prevalence rate among individuals who refused consent for the fixed effects, random effects and random effects bias correction models. Analytic standard errors are shown for the fixed effects and random effects models, with bootstrapped errors for random effects and random effects bias correction models using 1,000 replications. Also shown is the HIV rate using an imputation model for men who refused consent. Prevalence estimates are weighted and account for survey design. Source: DHS Zambia 2007 (men).

Table A7: Marginal Effects from a Probit Model for HIV Test Consent for Ghanaian Men

	HIV Test Consent	
Variables	Individual Level Variables	Interviewer Level Averages
Years of Education	0.00224*	-0.0002
	(0.001)	(0.000)
**Wealth Category**		
Poorest	0.09640***	0.0136**
	(0.016)	(0.007)
Poorer	0.08063***	0.0200***
	(0.014)	(0.001)
Middle	0.05346***	0.0417***
	(0.015)	(0.007)
Richer	0.03687***	0.0376***
	(0.014)	(0.006)
**Marital Status**		
Currently Married	0.02424	-0.0035
	(0.022)	(0.004)
Formerly Married	-0.04371	0.0084
	(0.028)	(0.011)
Had STD	0.03830*	0.0529***
	(0.021)	(0.009)
**Age at First Intercourse**		
Never Had Sex	0.00918	-0.0578***
	(0.021)	(0.009)
15 or Younger	-0.02929	-0.0569***
	(0.018)	(0.010)
**Had High Risk Sex**	0.00272	0.0092
	(0.019)	(0.007)
**Number of Partners**		
1	0.00304	-0.0331***
	(0.022)	(0.005)
2+	-0.00752	0.0218**
	(0.031)	(0.010)
Condom Last Intercourse	0.00045	-0.1715***
	(0.017)	(0.024)
Would Care for HIV Relative	0.02213*	0.0124***
	(0.013)	(0.001)
Know Someone Died of AIDS	0.01538	-0.0424***
	(0.010)	(0.006)
Previously HIV Tested	0.00564	-0.0097***

Table A7 – Continued on the Next Page

	HIV Test Consent	
Variables	Individual Level Variables	Interviewer Level Averages
	(0.017)	(0.003)
Smoker	-0.00928	-0.0233***
	(0.017)	(0.007)
**Language**		
Akan	0.04955***	-0.0147***
	(0.017)	(0.001)
Ga	0.06397**	0.0282***
	(0.026)	(0.005)
Ewe	0.02522	0.0175***
	(0.038)	(0.005)
Nzema	0.043	0.0112*
	(0.047)	(0.007)
Dagbani	0.01586	-0.0241***
	(0.049)	(0.007)
Other	-0.03509	
	(0.030)	
Observations	4,955	

Robust standard errors in parentheses
*** p <0.01, ** p <0.05, * p <0.1

Note to table A7: Age group, region, religion, and ethnicity are included in the regression but are not shown. Each column is for the same regression where HIV consent is regressed on the X variables and the corresponding average (in percent) for each interviewer. A probit model is used, and marginal effects (absolute change in the probability of a positive outcome) are shown. In some cases standard errors are very small, but not zero as appears in the table due to rounding. Interviewer averages of categorical variables are a proportion ranging from 0 to 1. Standard errors account for clustering at the PSU level.

Table A8: Marginal Effects for Heckman-type Selection Models (Ghana 2003)

Variables	Maximum Likelihood Fixed Effects Consent Equation	Maximum Likelihood Fixed Effects HIV Equation	Maximum Likelihood Random Effects Consent Equation	Maximum Likelihood Random Effects HIV Equation	Bias Correction Random Effects Consent Equation	Bias Correction Random Effects HIV Equation
Interviewer Effect				0.2125***		0.2120***
				(0.020)		(0.020)
Years of Education	0.002	0.0004	0.0023*	0.0004	0.0023*	0.0004
	(0.001)	(0.000)	(0.001)	(0.000)	(0.001)	(0.000)
**Wealth Category**						
Poorest	-0.0187	0.0039	-0.0184	0.0039	-0.0184	0.004
	(0.020)	(0.005)	(0.019)	(0.005)	(0.019)	(0.005)
Poorer	-0.0563***	0.0078	-0.0553***	0.0078	-0.0554***	0.0079
	(0.020)	(0.006)	(0.020)	(0.006)	(0.020)	(0.006)
Middle	-0.0747***	0.0017	-0.0769***	0.0017	-0.0769***	0.0018
	(0.021)	(0.006)	(0.021)	(0.006)	(0.021)	(0.006)
Richer	-0.1139***	0.0021	-0.1169***	0.0021	-0.1171***	0.0023
	(0.024)	(0.007)	(0.023)	(0.007)	(0.023)	(0.007)
**Marital Status**						
Currently Married	0.0241	0.0128	0.0235	0.0128	0.0237	0.0128
	(0.022)	(0.008)	(0.022)	(0.008)	(0.022)	(0.008)
Formerly Married	-0.0382	0.0088	-0.0420*	0.0087	-0.0418*	0.0088
	(0.024)	(0.008)	(0.024)	(0.008)	(0.024)	(0.008)
Had STD	0.0461	0.0093	0.0457	0.0094	0.0456	0.0094
	(0.029)	(0.007)	(0.028)	(0.007)	(0.028)	(0.007)
**Age at First Intercourse**						
15 or Younger	0.0502**	-0.0422	0.0485**	-0.0254	0.0487**	-0.0228
	(0.022)	(0.035)	(0.021)	(0.031)	(0.021)	(0.030)
>15	0.0374*	-0.0475	0.0356*	-0.0334	0.0357*	-0.0312
	(0.022)	(0.034)	(0.021)	(0.030)	(0.021)	(0.030)
**Had High Risk Sex**	0.0531*	-0.0275	0.0546**	-0.0199	0.0544**	-0.0184
	(0.027)	(0.025)	(0.027)	(0.022)	(0.027)	(0.021)
**Number of Partners**						
1	0.004	-0.01	0.0033	-0.01	0.0032	-0.0101
	(0.022)	(0.007)	(0.022)	(0.007)	(0.022)	(0.007)
2+	-0.0064	-0.0178*	-0.0068	-0.0180*	-0.0069	-0.0180*
	(0.031)	(0.011)	(0.030)	(0.011)	(0.031)	(0.011)
Condom Last Intercourse	0.0001	0.0083*	0.0000	0.0082*	0.0000	0.0083*
	(0.018)	(0.004)	(0.018)	(0.004)	(0.018)	(0.004)
Would Care for HIV Relative	0.0221*	-0.0007	0.0230*	-0.0007	0.0229*	-0.0007
	(0.013)	(0.004)	(0.012)	(0.004)	(0.012)	(0.004)
Know Someone Who Died of AIDS	0.0163	-0.0009	0.0162	-0.001	0.0161	-0.0009
	(0.011)	(0.003)	(0.011)	(0.003)	(0.011)	(0.003)
Previously HIV Tested	0.004	-0.0054	0.0046	-0.0054	0.0049	-0.0056
	(0.018)	(0.007)	(0.018)	(0.007)	(0.018)	(0.007)
Smoker	-0.0103	0.0057	-0.0089	0.0057	-0.0089	0.0058
	(0.017)	(0.004)	(0.017)	(0.004)	(0.017)	(0.004)
**Language**						
Akan	0.0474***	0.0042	0.0513***	0.0043	0.0511***	0.0044
	(0.017)	(0.004)	(0.016)	(0.004)	(0.016)	(0.004)
Ga	0.0766*	0.0009	0.0864**	0.001	0.0858**	0.001
	(0.045)	(0.013)	(0.044)	(0.013)	(0.044)	(0.013)
Ewe	0.0338	0.0122	0.0272	0.0122	0.0271	0.0123
	(0.045)	(0.009)	(0.045)	(0.009)	(0.045)	(0.009)
Nzema	0.0537	0.0253*	0.0484	0.0253*	0.0489	0.0254*
	(0.060)	(0.015)	(0.052)	(0.015)	(0.052)	(0.015)
Dagbani	-0.0201	-0.1142***	0.0187	-0.1387***	0.0186	-0.8298***
	(0.063)	(0.018)	(0.038)	(0.021)	(0.038)	(0.084)
Other	-0.0391	-0.0102	-0.0327	-0.0102	-0.0328	-0.0102
	(0.027)	(0.009)	(0.025)	(0.009)	(0.025)	(0.009)
Observations	4,955	4,955	4,955	4,955	4,955	4,955

Robust standard errors in parentheses
*** p <0.01, ** p <0.05, * p <0.1

Note to table A8: Controls for age group, region, ethnicity, and religion are included in each regression equation but not shown. The model is a bivariate probit for HIV status and consent to a HIV test. Data are for men who completed an interview. The first model uses interviewer fixed effects as the exclusion restriction. The coefficients from the interviewer effects are not shown in the table. The second model uses the random effects procedure where HIV consent is regressed on the X variables, along with the mean values for each interviewer (see table A3). The average error term for each interviewer is added to the predicted value of the interviewer means, and used as the exclusion restriction in the HIV regression. The final model is the random bias correction procedure using the same exclusion restriction. Coefficients are obtained by restricting the value of ρ to its random effects bias correction estimate and implementing the bivariate probit at that value. Marginal effects (absolute change in the probability of a positive outcome) are shown. Source: DHS Ghana 2003 (men). Standard errors account for clustering at the PSU level.

Table A9: Correlation Coefficient (ρ) Estimates for Men in Ghana

Model for Refused Consent	Parameter Value	Analytic 95% CI		Bootstrap 95% CI	
Fixed Effects Model	0.93	0.50	0.99		
Random Effects Model	0.93	0.02	1.00	0.56	1.00
Random Effects Bias Correction Model	0.59			0.39	0.72

Note to table A9: The table shows the estimated correlation coefficient between consent and HIV status for the fixed effects, random effects and random effects bias correction models. Analytic standard errors are shown for the fixed effects and random effects models, with bootstrapped errors for random effects and random effects bias correction models using 1,000 replications. Source: DHS Ghana 2003 (men).

Table A10: HIV Prevalence (%) among Men who Refused to Test in Ghana

Model for Refused Consent	Parameter Value	Analytic 95% CI		Bootstrap 95% CI	
Fixed Effects Model	1.00E-07	3.00E-08	2.00E-07		
Random Effects Model	2.00E-05	1.00E-06	4.00E-06	1.00E-08	0.16%
Random Effects Bias Correction Model	0.07%	0.05%	0.09%	0.03%	0.35%
Imputation Model	1.82%	1.60%	20.48%		

Note to table A10: The table shows the estimated HIV prevalence rate among individuals who refused consent for the fixed effects, random effects and bias correction models. Analytic standard errors are shown for the fixed effects and random effects models, with bootstrapped errors for random effects and bias correction models using 1,000 replications. Also shown is the HIV rate using an imputation model for men who refused consent. Estimates lower than 0.01% are shown in scientific notation. Prevalence estimates are weighted and account for survey design. Source: DHS Ghana 2003 (men).

Additional Figures

Figure A1: Histogram of Number of Interviews by Interviewer in Zambia 2007 (Men)

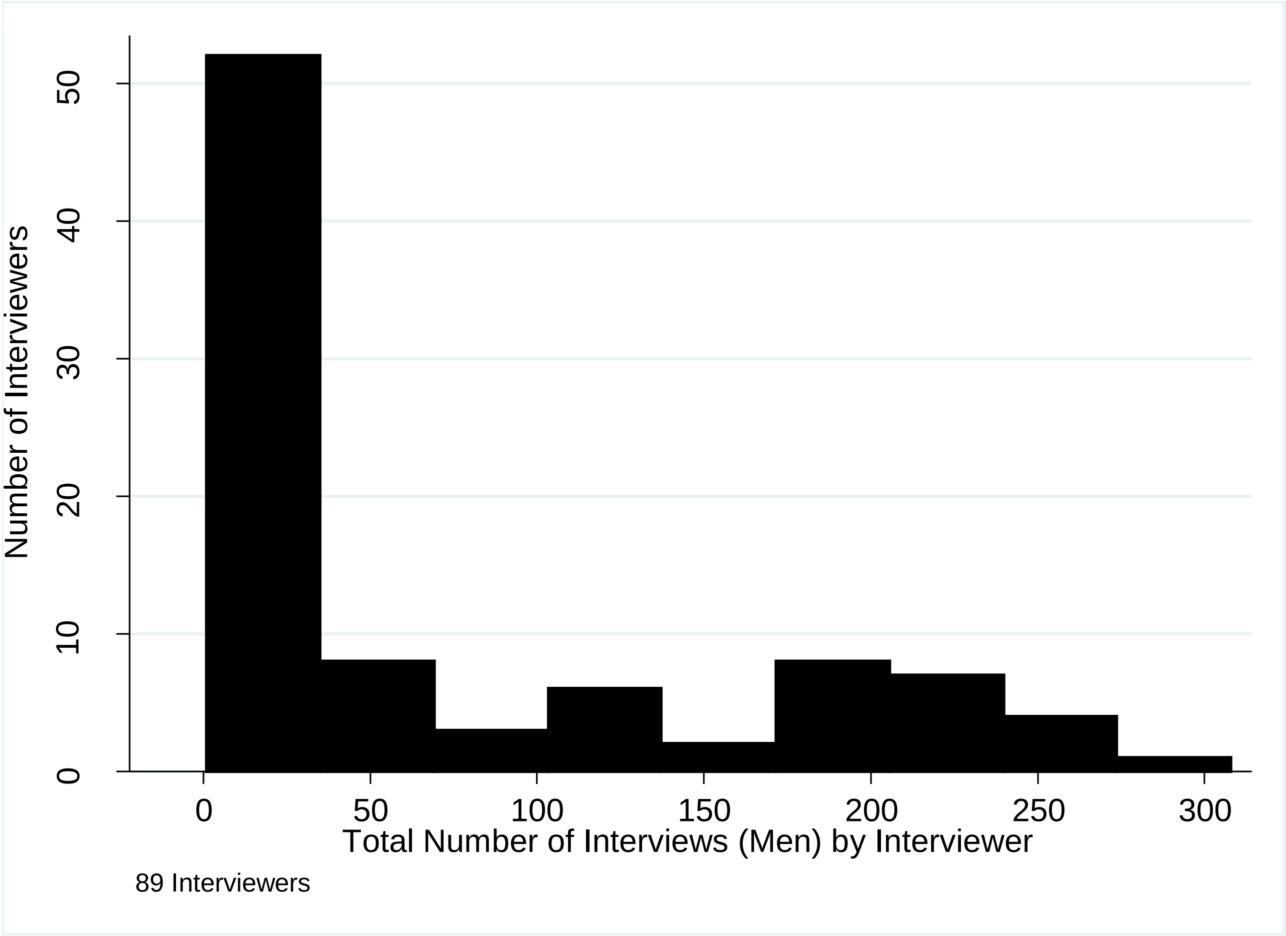

Graph is at the interviewer level (one observation per interviewer).

Figure A2: Histogram of Consent Rates by Interviewer in Zambia 2007 (Men)

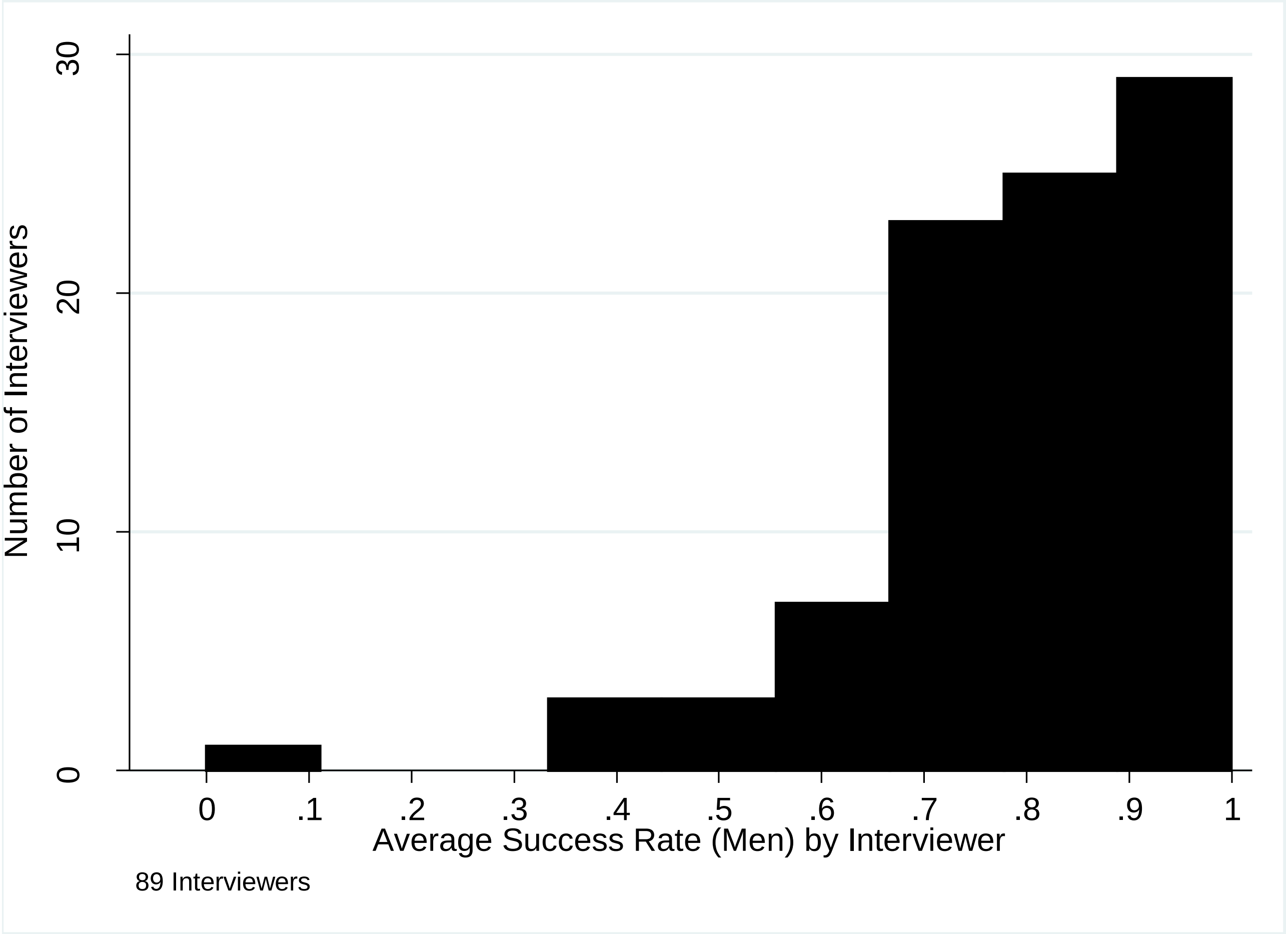

Note to Figure A2: Graph is at the interviewer level (one observation per interviewer). For each interviewer, their consent rate is calculated as the number of respondents from whom consent to test for HIV was obtained by the interviewer, divided by the number of respondents from whom consent to test for HIV was sought by the interviewer.

Figure A3: Histogram of Number of Interviews by Interviewer in Ghana 2003 (Men)

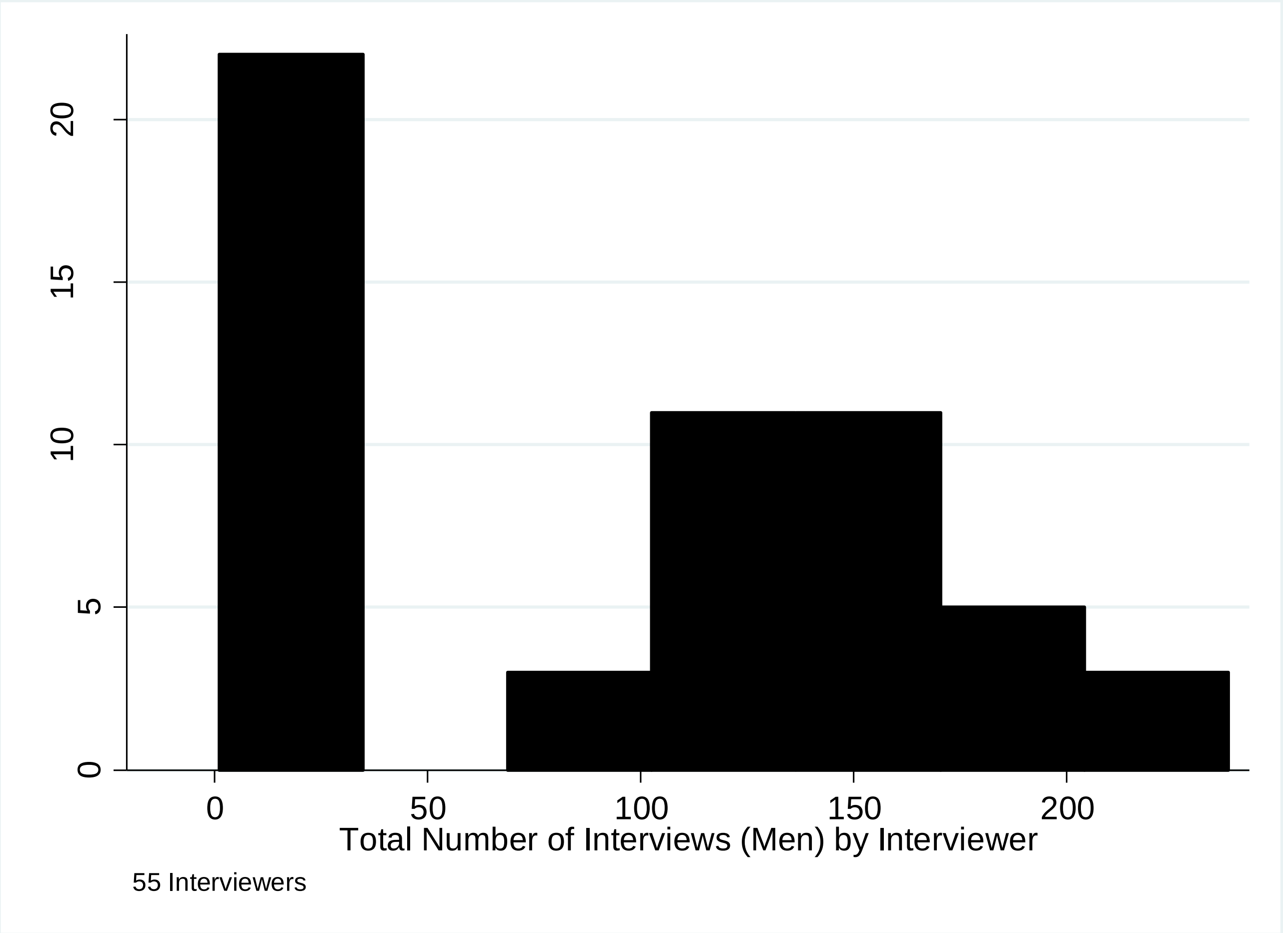

Graph is at the interviewer level (one observation per interviewer).

Figure A4: Histogram of Consent Rates by Interviewer in Ghana 2003 (Men)

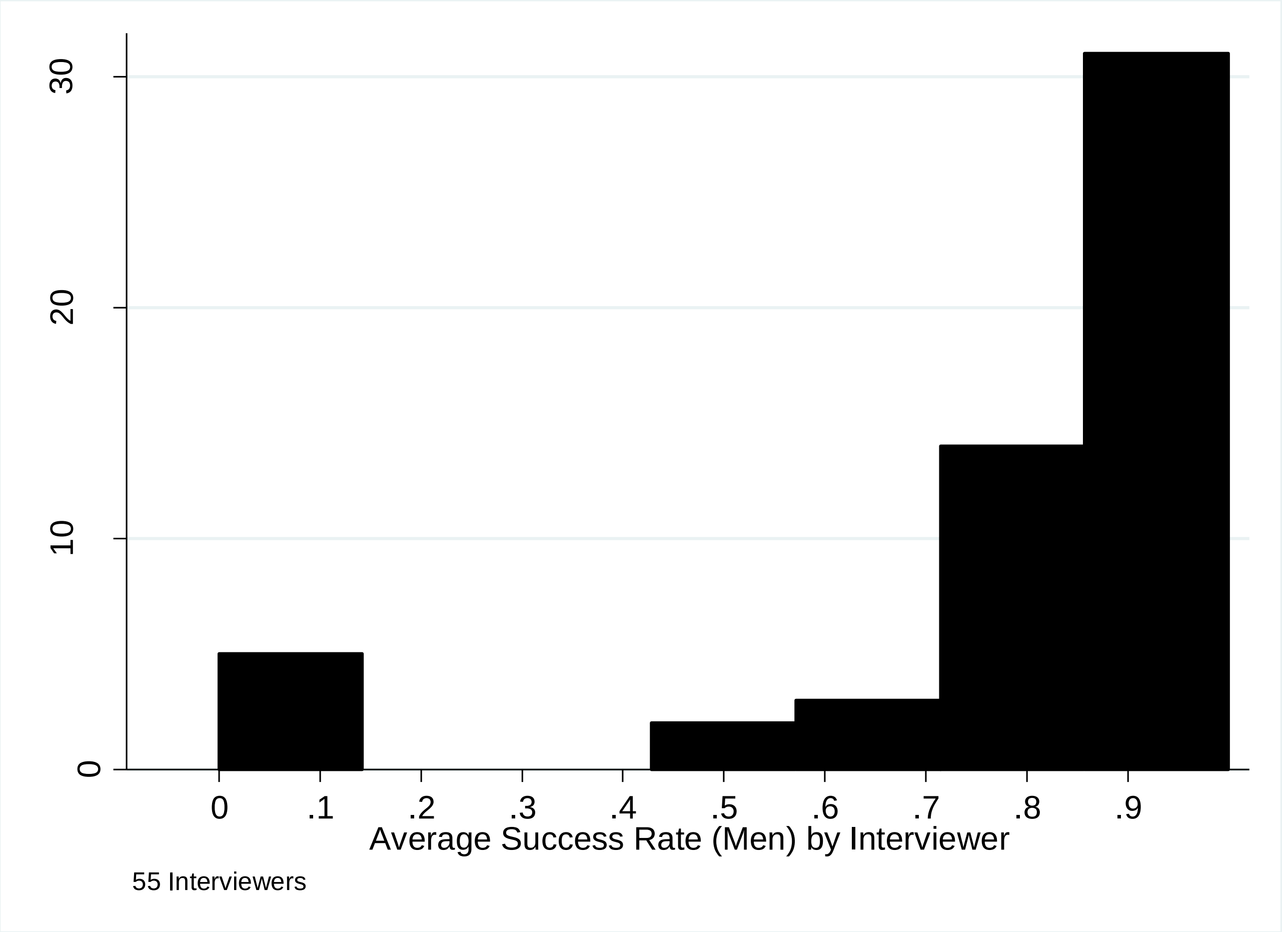

Note to Figure A4: Graph is at the interviewer level (one observation per interviewer). For each interviewer, their consent rate is calculated as the number of respondents from whom consent to test for HIV was obtained by the interviewer, divided by the number of respondents from whom consent to test for HIV was sought by the interviewer.

References

1. W. P. Van de Ven and B. Van Praag. The demand for deductibles in private health insurance: A probit model with sample selection. *J Econom*, 17:229–252, 1981.

2. J. A. Dubin and D. Rivers. Selection bias in linear regression, logit and probit models. *Sociol Methods Res*, 18:360–390, 1989.

3. T. Bärnighausen, J. Bor, S. Wandira-Kazibwe, and D. Canning. Correcting HIV prevalence estimates for survey nonparticipation using Heckman-type selection models. *Epidemiology*, 22:27–35, 2011.

4. D. Madden. Sample selection versus two-part models revisited: the case of female smoking and drinking. *J. Health Econ.*, 27:300–307, 2008.

5. Y. Mundlak. On the pooling of time series and cross section data. *Econometrica*, 46:69–85, 1978.

6. G. Chamberlain. Analysis of covariance with qualitative data. *Rev Econ Stud*, 47:225–238, 1980.

7. K. M. Murphy and R. H. Topel. Estimation and inference in two-step econometric models. *J Bus Econ Stat*, 20:88–97, 2002.

8. J. S. Butler. Estimating the correlation in censored probit models. *Rev Econ Stat*, 78:356–358, 1996.

9. R. C. Chiburis, J. Das, and M. Lokshin. A practical comparison of the bivariate probit and linear IV estimators. *Econ Lett*, 117:762–766, 2012.

10. D. W. Andrews. Estimation when a parameter is on a boundary. *Econometrica*, 67:1341–1383, 1999.

11. D. W. Andrews. Inconsistency of the bootstrap when a parameter is on the boundary of the parameter space. *Econometrica*, 68:399–405, 2000.

12. W. Greene. The behaviour of the maximum likelihood estimator of limited dependent variable models in the presence of fixed effects. *Econom J*, 7:98–119, 2004.

13. S. Greenland. Small-sample bias and corrections for conditional maximum-likelihood odds-ratio estimators. *Biostatistics*, 1:113–122, 2000.

14. S. A. Murphy and A. W. Van der Vaart. On profile likelihood. *J. Am. Stat. Assoc.*, 95: 449–465, 2000.

15. D. J. Corsi, M. Neuman, J. E. Finlay, and S. Subramanian. Demographic and health surveys: a profile. *Int. J. Epidemiol.*, 41:1602–1613, 2012.

16. D. R. Hogan, J. A. Salomon, D. Canning, J. K. Hammitt, A. M. Zaslavsky, and T. Bärnighausen. National HIV prevalence estimates for sub-saharan Africa: controlling selection bias with Heckman-type selection models. *Sex. Transm. Infect.*, 88:i17–i23, 2012.
